# Trafficking of lymphocytes into the CNS

**DOI:** 10.18632/oncotarget.5014

**Published:** 2015-07-17

**Authors:** Nicholas Schwab, Tilman Schneider-Hohendorf, Heinz Wiendl

**Affiliations:** Department of Neurology, University of Muenster, Muenster, Germany

**Keywords:** Pathology Section

Immune cell migration over the blood-brain barrier into the central nervous system has been a topic of research for the last century, but it has gained significant momentum 20 years ago with the possibility of blocking adhesion molecules with monoclonal antibodies and use these as a therapeutic avenue for immune-mediated tissue damage (Yednock et al., 1992). In principle this concept applies to organ transplantation, as well as to autoimmunity, disease examples being psoriasis, Crohn's disease, and multiple sclerosis (Stüve and Wiendl, 2009). The general principle is easy: immune cells are equipped with a set of (surface) molecules, which they use to interact with the respective ligands on the surface of the endothelial barriers separating blood circulation from the central nervous system tissue. Progress in immunology of the last few years has contributed numerous novel immune cell subpopulations and deep-resolution characterization of know subsets, all better characterized by different sets of e.g. cell surface molecules, cytokine production or transcription signatures (Ransohoff, 2009). To complicate matters even more, the field now acknowledges the existence of at least 3-4 “blood-brain barriers” or trafficking routes into the CNS, each of them comprising a different set of endo- and epithelial cells with their own and unique expression of (adhesion) molecules. Furthermore, homeostatic and pathological conditions can change those patterns completely, e.g. during inflammation (Engelhardt and Ransohoff, 2012). Cellular adhesion of lymphocytes under physiological flow conditions depends on several steps (activation, rolling, weak- and firm-adhesion, crawling, diapedesis) even though these steps can differ between cellular subsets and the respective barrier.

Molecular interactions between PSGL-1 and P-/E-/L-selectin, LFA-1 and ICAM-1/2, and especially VLA-4 and VCAM-1 have been the main focus of immune cell adhesion. However, to find out exactly which immune cells use which molecules to interact with which molecule on the barrier side is a herculean task. Natalizumab, a blocking monoclonal antibody against the integrin alpha chain of VLA-4, works by completely blocking the VLA-4-mediated adhesion step of lymphocytes to endothelial cells and has been proven to be a very effective treatment in most MS patients. However, if the next generation of treatments against immune-mediated diseases is supposed to be more specific in its mechanisms of action, it is imperative to functionally elucidate exactly these interactions. Our work about CD4 cells and their expression of VLA-4, PSGL-1, and MCAM contributes one facet to the overall picture (Schneider-Hohendorf et al., 2014). We could show that if human T cells are subjected to *in vivo* VLA-4 blockade (during treatment with natalizumab), they continuously upregulate PSGL-1 and can use it to enhance their rolling capacities over primary human endothelial cells. As this „upregulation“ takes months and years to reach a plateau, we speculate that it is not in fact a gene regulation process, but more a process on a population level, in which the immune system over time selects for those T cells with higher PSGL-1 expression, leading to a gradual increase of this molecule in the overall population. However, these T cells might be able to roll alongside the endothelium with a higher frequency (and also on endothelial cells with lower expression of PSGL-1 ligands than cells without that upregulation), but without VLA-4 they are unable to adhere to it. At least this is true for most T cells. Interestingly, there is an adhesion molecule, which was originally discovered during oncological studies: MCAM (melanoma cell adhesion molecule). A subpopulation of T cells express MCAM (it has been shown that these cells are mostly Th17 cells) and we could show that they can use their MCAM expression to adhere to endothelial cells even without using VLA-4. *In vivo* this leads to the CSF-resident cells of natalizumab-treated patients to express no VLA-4 and high quantities of MCAM. Whether these MCAM+ cells use an MCAM-ligand interaction to mediate adhesion or if they use MCAM to mediate adhesion by proxy, because they possess a functional advantage due to their expression of this molecule, is still unclear. The MCAM+ cells in the CSF and the CNS of these patients are obviously unable to trigger relapses on their own, as all Th1 cells depend on VLA-4 to enter the CNS and this molecule is blocked by natalizumab. However, even without relapses it is possible that these cells can mediate some form of damage on their own and contribute to underlying slow processes of disease progression. Additionally, this might be the reason why the cessation of natalizumab usually leads to a strong rebound of MS activity (Melis et al., 2013): as the relapse-triggering cells (Th17) are already located in the CNS, the removal of the VLA-4 blockade allows for a swift and immense recruitment of Th1 cells. Future studies will have to explore the possibility of treating MS patients according to their pathogenic phenotype: Th1-driven MS pathology might be effectively treated with blockade of VLA-4, but Th17-driven pathology probably will not. As Th17 cells migrate (over the choroid plexus) into the CNS using a different set of adhesion molecules (Rothhammer et al., 2011), a different treatment might be better suited to inhibit their pathological potential. Whether that is the combined blockade of VLA-4 and MCAM remains to be seen, as these cells might also fulfill important functions in immune surveillance that we do not yet understand and the complete blockade of CNS infiltration could lead to even more severe adverse events such as PML.

In summary, trafficking lymphocytes and specifically lymphocyte subpopulations have individual signatures of adhesion molecules that allow for entry into the CNS. Neutralization of VLA-4 with the monoclonal antibody natalizumab, a highly effective therapy for MS, does not completely prevent all leukocyte traffic into the CNS. It does, however, inhibit central memory cells from entering and overall induces a dramatic, but reversible shift of the lymphocytes and immune cell milieu in the CNS and the CSF. This might explain, why in the general MS population the lack of migration inhibition of Th17 cells is without major clinical consequences, but in some forms of inflammatory CNS diseases (such as e.g. the NMO spectrum) this therapy does not work.

